# A census of the edition of 1555 of Andreas Vesalius' *De Humani Corporis Fabrica*

**DOI:** 10.1186/1755-7682-2-26

**Published:** 2009-09-08

**Authors:** Stephen N Joffe

**Affiliations:** 1 The Medical History Institute, The Joffe Foundation, 4400 Drake Road, Cincinnati, OH 45243, USA

## Abstract

The purpose of this study was to determine the locations of the second edition (1555) of the De Humani Corporis Fabrica written by Vesalius.

Contacts were made with institutions of higher learning, museum libraries, and libraries of national collections, libraries of research institutions, cathedral libraries, antique book dealers, trade journals, book auctions and private collectors.

A total of 113 copies of the 1555 *Fabrica *were found in University and Institutional Libraries. Of them, 33 (29%) were in the United Kingdom; 35 (31%) in Europe and 45 (40%) in the USA. Location of the second edition Vesalius in private collections was more difficult to objectively determine and accounts for approximately 10% of the second Edition books in the census.

## Introduction

In 1555, the Belgian anatomist Andreas Vesalius, together with his publisher, Johannes Oporinus of Basel, produced a second folio edition of this revolutionary work *De Humani Corporis Fabrica*.

Cushing included an incomplete list of holdings of all Vesalian works that he traced as part of this *Bio-bibliography of Andreas Vesalius*, what he calls an "Index of Recorded Copies [1] A later census of the 1543 edition, compiled by Horowitz and Collins and published in 1984, was created as a result of their research into variant variant copies of that edition[2] In 1994, Elly Cockx-Indestege published the results of her research into every copy of every pre-1800 edition of all Vesalius' works held in Belgian collections, including five copies of the 1555 edition [1].

This report presents a list of copies of the second edition of (1555) De Humani Corporis written by Vesalius and their institutional locations 450 years since publication.

## Materials and methods

A great boon to the 21^st^-century researcher is the development of the internet. The number of university and institutional libraries which are searchable online means that the libraries of over 200 institutions could be searched in a matter of several months.

While these innovations have undoubtedly shortened dramatically the amount of time researchers have to spend physically turning the pages of printed catalogues, the "detective techniques" used by Owen Gingerich in his *Great Copernicus Chase *still provide the mainstay of this research. Many libraries were not yet online, or are in the process of being made available online. Letters of enquiry, consultation with dealers, advertising in bibliophilic journals, and systematic checking through the multivolume Book Auction Records and original auction catalogues have therefore all been vital to the collection and collation of the information in this catalogue.

The method of researching copies held in institution libraries was relatively straightforward. Once it had been ascertained which institutions owned copies of the 1555 edition, requests were sent to the relevant librarians for any bibliographical details that did not appear in their online catalogues. In some cases, such further information was not available and wherever possible, the original copy was then examined in person in the United Kingdom. That this catalogue is so complete is due in the main to those librarians, who went out of their way to help this research, both through their own investigations, suggestions and comments, and the ability to cross-reference information provided.

Discovering copies held in private collections was a more involved process. Auction houses were naturally discreet with regard to their clients' identities; records from the earlier sales often do not survive, and catalogue descriptions from pre-1980s catalogues tend to be brief.

While recent catalogues provide a great deal of detailed bibliographic information, those dating from earlier than the mid-seventies do not supply much information beyond basic notes on the completeness and condition of the copy. This brevity of description makes it difficult to identify the copy in question, which in turn means that it is difficult to guarantee that the same copy, sold in different sales in different countries was not counted as two separate copies. Unfortunately, unless an owner, knowing the provenance of his or her copy, recognizes it on the list of those sold at auction, there is no way of remedying this particular problem.

The Book Auction Records for the past 100 years have been searched, and it has sometimes been possible to trace the movement of a single copy from sale to sale. Catalogues for individual sales have also been looked at, though even those containing the auctioneer's own notations usually give not further clue to the provenance, seller or buyer of the copy in question. Even those auction houses that are still in business are unlikely to be in possession of any further information. As in the case of Southerans, whose records were destroyed in the Blitz of the Second World War, records often just no longer exist.

Although the chronological scope of the catalogue was originally intended to cover the past century, the lack of information available has meant that though sales from the early 20^th ^century have been included, it is only in a very few cases that the present day whereabouts of those copies is known.

For more recent sales, Christie's and Sotheby's, as the auction house through which the vast majority of more recent copies have been sold, have been exceptionally helpful. Within the boundaries of their client confidentiality, both companies have enabled the present owners of most copies to be established.

Auction and dealer sales in the United Kingdom were examined from the year 1900 to 2006. The copies were then tabulated to report as accurately as possible the copies known to currently exist in both private and public collections. Those books in private collections were personally reviewed or third party verified.

## Results

Results are presented as a geographic list of holdings within the United Kingdom Europe (Table 1 and 2) and the USA (Table 3). As well as complete works, there are some instances where fragmentary copies (usually comprising solely of the illustrations) are held. These have been excluded in the main body of the census. Also provided is the list of copies known to have been sold through auction houses and dealers during the last century (Table 4). These copies are placed in the chronological order of their last appearance on the market. The last known location is given for these privately held copies. It is certain that in many cases the copy is now held somewhere else.

**Table 1:** Copies held in University and Institution Libraries in the United Kingdom (UK)

I. ABERDEEN	Aberdeen University Special Libraries and Archives
2. BIRMINGHAM	Central England University
3. CAMBRIDGE	Cambridge University, Caius College Library
4. CAMBRIDGE	Cambridge University, Emmanuel College Library
5. CAMBRIDGE	Cambridge University, St John's College Library
6. CAMBRIDGE	Cambridge University Library, Copy 1
7. CAMBRIDGE	Cambridge University Library, Copy 2
8. CAMBRIDGE	Cambridge University School of Anatomy Library
9. DURHAM	Durham University, Palace Green Library
10. EDINBURGH	Edinburgh University Library, Copy 1
11. EDINBURGH	Edinburgh University Library, Copy 2
12. EDINBURGH	The Royal College of Physicians Edinburgh
13. EXETER	Exeter Cathedral Library
14. GLASGOW	Glasgow University, Glasgow University Library Special Collections
15. LIVERPOOL	Liverpool University, Sydney Jones Library Special Collections
16. LONDON	The British Library
17. LONDON	University College London Special Collections, Copy 1
18. LONDON	University College London Special
19. LONDON	National Art Library, Victoria and Albert Museum
20. LONDON	Royal Academy of Arts Library
21. LONDON	The Royal College of Obstetricians and Gynaecologists
22. LONDON	The Royal College of Physicians London
23. LONDON	The Royal Society
24. LONDON	The Science Museum
25. LONDON	Wellcome Trust Library History of Medicine Collections
26. MANCHESTER	Manchester University, John Rylands Library Special Collections, Copy 1
27. MANCHESTER	Manchester University, John Rylands Library Special Collections, Copy 2
28. NEWCASTLE	Newcastle University, Robinson Library Special Collection
29. OXFORD	Oxford University Bodleian Library, Copy 1
30. OXFORD	Oxford University Bodleian Library, Copy 2
31. OXFORD	Oxford University, Corpus Christi Library
32. READING	Reading University, Whiteknights Library
33. WELLS	Wells Cathedral Library

**Table 2:** Copies held in University and Institution Libraries in Europe (Excluding the UK)

1. AARHUS	Aarhus Technical Library
2. AARHUS	State and University Library
3. AUGSBURG	State Library of Augsburg
4. BAYERN	Bayerische Staatsbibliothek
5. BERGEN	University of Bergen
6. BOLOGNA	University of Bologna
7. COLOGNE	German National Library of Medicine
8. COPENHAGEN	Danish National Library of Science and Medicine
9. DUBLIN	Royal College of Surgeons
10. ERLANGEN	University of Erlangen-Nurnberg
11. HALLE	Goettingen State and University Library
12. HALLE	University of Halle, Copy 1
13. HALLE	University of Halle, Copy 2
14. HALLE	German Academy of Naturforscher
15. HELSINKI	Helsinki University
16. JENA	University of Jena, Copy 1
17. JENA	University of Jena, Copy 2
18. LIVORNO	Communal Library
19. MADRID	University of Madrid, Copy 1
20. MADRID	University of Madrid, Copy 2
21. MADRID	University of Madrid, Copy 3
22. MUNICH	University of Munich
23. OSLO	Bibliotekformedisinoghelsefag
24. OSLO	Bibliotek for Medicine
25. PADOVA	Library of Seminario Maggiore
26. PADOVA	University of Padova
27. ROME	Biblioteca Casanatense
28. ROME	Biblioteca Medica Statale
29. ROME	University of Sassari
31. VIENNA	Osterreichische Nationalbibliothek
32. WOLFENBUTTEL	Herzog Anton Library, Copy 1
33. WOLFENBUTTEL	Herzog Anton Library, Copy 2
34. WOLFENBUTTEL	Herzog Anton Library, Copy 3
35. ZURICH	University of Zurich

**Table 3:** Copies in University and Institution Library in United States of America (US)

1. ALBANY, NY	New York State Library
2. ANN ARBOR, MI	University of Michigan, Copy 1
3. ANN ARBOR, MI	University of Michigan, Copy 2
4. AUSTIN, TX	University of Texas
5. BALTIMORE, MD	The Johns Hopkins University, Copy 1
6. BALITMORE, MD	The Johns Hopkins University, Copy 2
7. BALTIMORE, MD	National Library of Medicine
8. BOSTON, MA	Boston University
9. BOSTON, MA	Boston Public Library
10. CAMBRIDGE, MA	Dibner Institute for the History of Science and Technology
11. CAMBRIDGE, MA	Harvard University, Copy 1
12. CAMBRIDGE, MA	Harvard University, Copy 2
13. CAMBRIDGE, MA	Harvard University, Copy 3
14. CAMBRIDGE, MA	Harvard University, Copy 4
15. CHARLOTTSVILLE, VA	University of Virginia, Copy 1
16. CHARLOTTSVILLE, VA	University of Virginia, Copy 2
17. CHICAGO, IL	Northwestern University
18. CHICAGO, IL	University of Illinois
19. CLEVELAND, OH	Case Western Reserve University
20. COLUMBUS, OH	Ohio State University
21. DALLAS, TX	University of Texas SW Medical Centre
22. DENVER, CO	University of Colorado
23. HANOVER, NH	Dartmouth College
24. IOWA CITY, IA	University of Iowa
25. KANSAS CITY, KS	University of Kansas Medical Center
26. LOS ANGELES, CA	University of California, Copy 1
27. LOS ANGELES, CA	University of California, Copy 2
28. MINNEAPOLIS, MN	University of Minnesota
29. NASHVILLE, TN	Vanderbilt University, Copy 1
30. NASHVILLE, TN	Vanderbilt University, Copy 2
31. NEW HAVEN, CT	Yale University, Copy 1
32. NEW YORK, NY	New York Academy of Medicine
33. OKLAHOMA CITY, OK	University of Oklahoma
34. OMAHA, NE	University of Nebraska
35. PHILADELPHIA, PA	Thomas Jefferson University
36. PHILADELPHIA, PA	University of Pennsylvania, Copy 1
37. PHILADELPHIA, PA	University of Pennsylvania, Copy 2
38. PHILADELPHIA, PA	College of Physicians of Philadelphia, Copy 1
39. PHILADELPHIA, PA	College of Physicians of Philadelphia, Copy 2
40. PHILADELPHA, PA	College of Physicians of Philadelphia, Copy 3
41. PITTSBURGH, PA	University of Pittsburgh
42. PORTLAND, OR	Oregon Health Science University
43. ROCHESTER, NY	University of Rochester
44. SALT LAKE CITY, UT	University of Utah
45. WASHINGTON, DC	Institute of Medicine
**CANADA**	
1. VANCOUVER BC, CANADA	University of British Columbia

**Table 4:** United Kingdom (UK) auction and dealer sales 1900- To Date (July 2009)

1. 2003:	H.M. Fletcher, Hertfordshire, Location: UK Private
2. 2002	Quaritch, London, Location: UK Private
3. 2001	WP Watson, London, Location: UK Private
4. 20.10.99	Christie's London, Location: Private
5. 26.11.97	Christie's London, Location: Bought by US dealer, Martyan Lan Inc, New York in partnerships with UK dealer, Rick Watson and later sold.
6. 5.12.96	Sotheby's London, Location: Sold to a Spanish dealer
7. 31.3.95	Christie's South Kensington, Location: UK private
8. 11.11.94	Sotheby's London, Location: US private, Goodrich
9. 18.5.89	Sotheby's London, Location: UK private
10. 16.11.88	Christie's London, Location: Sold to UK deal, Pickering & Chatto, now UK private
11. 14.4.88	Sotheby's London, Location: US, Boston University Library
12. 2.11.81	Sotheby's London, Location: ? Bennett
13. 17.6.81	Sotheby's London, Location: UK dealer, Quaritch (no further records available)
14. 17.6.68	Sotheby's London, Location: UK dealer, Dawson
15. 17.10.67	Sotheby's London, Location: US dealer, HS Levinson
16. 9.11.60	Sotheby's London, Location: UK dealer, Weil
17. 21.7.59	Sotheby's London, Location: UK dealer, Dawson
18. 21.5.58	Christie's London, Location: UK dealer, Weil (Later offered for sale in Weil Cat 28, no. 256)
19. 1950s	Simon Weil, London, Cat 22 no. 293, Location: Unknown
20. 16.7.51	Sotheby's London, Location: UK, HM Stationery Office
21.26.7.49	Sotheby's London, Location: UK dealer, Weil
22. 12.5.48	Hodgson and Son London, Location: UK dealer, King
23. 11.5.48	Sotheby's London, Location: UK dealer, Southeran (records since destroyed)
24. 19.3.47	Sotheby's London, Location: UK dealer, Edwards
25. 26.1.45	Hodgson and Son London, Location: UK dealer, Robinson
26. 30.3.44	Hodgson and Son London, Location: UK dealer, HK Lewis
27. 9.5.44	Sotheby's London, Location: UK dealer, Edwards
28. 31.3.43	Dowell's Edinburgh, Location: Unknown
29. 10.6.41	Sotheby's London, Location: UK dealer, Edwards
30. 6.2.34	Sotheby's London, Location: UK dealer, Duke
31.23.7.34	Sotheby's London, Location: UK dealer, Edwards
32. 1.6.31	Sotheby's London, Location: UK dealer, Dave and Orioli (Later offered for sale in Davies and Orioli Cat 132, no. 236)
33. 15.5.30	Hodgson and Son London, Location: UK dealer, Salamander
34. 14.5.25	Hodgson and Son London, Location: UK dealer, Lubrano
35. 17.3.24	Sotheby's London, Location: UK dealer, Edwards
36. 20.12.22	Hodgson and Son London, Location: UK dealer, Leighton
37. 31.7.13	Sotheby's London, Location: UK dealer, Maggs
38. 21.6.10	Sotheby's London, Location: UK dealer, Sawyer
39. 24.6.09	Hodgson and Son London, Location: Unknown
40. 5.12.06	Sotheby's London, Location: ?, Hozier
41. 12.7.06	Christie's London, Location: Private, Kirk

Twenty one of the copies sold at auction were subsequently bought by dealers of which nineteen (90%) were UK dealers. Eleven books are definitely in private collections today; another three were initially bought privately but could not be found.

This means of 74 books recorded as being present in the UK over the last century, only 42 are known to still be held there, of which 33 are confirmed to be at Universities or Public Institutions and at least nine are in private collections. The remaining 32 second edition Vesalius are probably now in private and university collections in the USA and Europe and are included in the Tables.

Eleven copies are confirmed to be privately owned by direct inspection or third party verification.

Due to security risks, privacy and the desire for anonymity, it was difficult to document additional books. Most of the books previously owned privately have been donated to University and Institutions, especially in the USA due to the tax benefits obtained by the donor.

## Discussion

Both Cushing and Cockx-Indestege's research took over 20 years to complete and despite the limiting of this census to one edition of one of Vesalius' works, it is till a huge undertaking [1,3].

The development of the internet is a great boon to the 21^st^-century researcher. University and institutional libraries are now searchable online; libraries of over 200 institutions could be rapidly searched. These innovations have shortened the time researchers need to spend physically turning the pages of printed catalogues.

Harvey Cushing, in his *Bio-bibliography of Andreas Vesalius*, recorded 25 copies in the US and only four copies in the UK of the first edition. This article presents the results of an attempt to enumerate and list the location of second edition copies.

The 1555 edition was more sumptuous than the 1543 first edition. It was printed on thicker paper, set in larger type and had more widely spaced lines. Vesalius made both stylistic and factual changes, and in some cases this required the design and production of a new initial letter woodblock. The new illustrations, with the exception of the title page, are generally considered to be even finer than those in the 1543 edition.

This second edition also had several textual alterations, including a revised chapter on embryology, a description of the venous valves, and two new chapters. No documentary evidence remains for the decision behind the production of a second edition except possibly to answer specific criticisms of the content leveled at the first edition and for Vesalius to answer his detractors in the new edition.

In addition, the market for the Fabrica remained strong as evidenced by the production in Lyons of an unauthorized cheaper pocket edition in 1552, as soon as the protective privilege granted to Vesalius by the French king had expired. This suggests that during the early 1550s, about the time that Vesalius and Oporinus began planning their second edition, demand for an expensive illustrated second version of the Fabrica remained high enough to make the effort and financial outlay of its production worthwhile.

An ideal starting point for a census would be to determine how many copies of the edition were originally printed. In the case of the 1555 *Fabrica*, however, the question cannot be answered conclusively as there are no surviving records of the print-run.

Records do survive from a printing house contemporary with that of Oporinus, though in a different country. Christoffel Plantjin or Plantin, as he is more familiarly known (1514-89), opened his printing house in Antwerp in the same year that the second edition of the *Fabrica *was produced. Plantin and his family kept detailed records of their business transactions, the survival of which is considered so rare that in 2001 the records were included in the UNESCO Memory of the World Register.

Then, as now, the number of copies to be printed of a book was decided by its potential market. Some editions were printed to order, and these ranged from 12-120 copies. However, as Leon Voet points out in *The Golden Compasses*, Plantin's son-in-law made it plain that such small runs did not make good financial sense, and editions were usually much larger. Although print-runs were flexible and could be altered according to his anticipation of the market, Plantin usually produced runs of 1,250 for ordinary editions, while works in great demand could be produced in editions of up to 2,500. However, most medical treatises, among certain other categories of book, were produced in runs of only 800.

From this evidence, it would seem likely the 1543 print run of the *Fabrica*, with its extraordinarily complex and numerous illustrations, would have been produced in numbers from the lower end of that scale, say about 800-1,000, and that the 1555 edition was also produced in similar numbers.

## Conclusion

This article presents in list form 113 copies of Andreas Vesalius' 1555 edition of De Humani Corporis Fabrica known to be held worldwide. Over the last century, a total of 74 second edition Vesalius' are recorded as having been in the UK. Only 42 copies are definitely now in the UK, with 33 in University and Institution Libraries. A further 35 (31%) of second edition books are in Europe and 45 (40%) in the USA.

It is estimated that over the last 450 years, between 10-15% of the 1555 edition of De Humani Corporis have survived and of these, the majority (90%) are in University and Public Institutional Libraries with very few now remaining in private collections.

## Competing interests

The author declares that they have no competing interests.
